# Hyponatremia severe and symptomatic in a critically ill infant

**DOI:** 10.1186/cc10208

**Published:** 2011-06-22

**Authors:** CMF Mangia, RM Sousa, AP Loretti, AFCF Martins, NF Oliveira, EL Lima, MC Andrade

**Affiliations:** 1 Universidade Federal de São Paulo, São Paulo - SP, Brazil

## Objective

To report a case of severe symptomatic hyponatremia secondary to previously undiagnosed congenital adrenal hyperplasia.

## Case

A 37-day-old infant, born at 38 weeks gestation, presented with hypoactivity, weight loss, poor feeding and vomiting in the hospital. The main clinical features were irritability, dehydration, hyponatremia, hyperkalemia and ambiguous genitalia. The biochemical data are presented in Table 1. The patient received isotonic fluids (first day) and treatment for severe chronic hyponatremia (developing over more than 48 hours) calculated to 125 mEq/l under slow correction in 96 hours. The sodium levels did not exceed 0.5 mEq/l/hour or 12 mEq/l/day. On the first day was initiated hydrocortisone (100 mg/m^2^) and afterwards 50 mg/m^2^. There were no complications of treatment and the child was discharged 2 weeks later without sequels. The karyotype was 46,XX.

**Table 1:** Serum electrolyte concentration during the first week

mEq/l	Admission	1st day	2nd day	3rd day	4th day	5th day	6th day	7th day
Na^+^	101	108	120	122	133	129	127	130
K^+^	7.9	6.1	4.7	3.3	4.5	5.8	5.3	5.6
Cl^-^	78	84	94	96	103	95	95	94

## Conclusion

Hyponatremia is a frequent electrolyte disorder. It is considered severe (<115 mEq/l) and chronic when the duration is >48 hours or the installation time is unknown. Irreparable harm can happen when abnormal serum sodium levels are corrected too quickly or too slowly. The correct diagnosis and understanding of the pathophysiology and mechanisms associated with hyponatremia allows establishing safe treatment criteria and consequently avoiding the sequels.
